# The effect of targeting and interceptive timing tasks on the brain waves of elite and educated athletes

**DOI:** 10.1371/journal.pone.0321539

**Published:** 2025-07-23

**Authors:** Fatemeh Keshvari, Alireza Farsi, Behrouz Abdoli, Alireza Bahrami

**Affiliations:** 1 Department of Cognitive and Behavioral Sciences and Technology in Sport, Faculty of Sport Sciences and Health, Shahid Beheshti University, Tehran, Iran; 2 Department of Motor Behavior and Sports Psychology, Faculty of Sport Sciences, Arak University, Arak, Iran; Shahid Chamran University of Ahvaz, IRAN, ISLAMIC REPUBLIC OF

## Abstract

The non-sports studies have separated two processing systems using paradigms of the roles of frontal regions in top-down processing, and the roles of parietal areas in bottom-up processing. However, the dynamics of cortical activity of the two processing systems in exercise have been neglected. This study examined the effect of targeting and interceptive timing tasks on brain waves in 20 participants, including 10 elite athletes and 10 educated individuals. Data were extracted using a 32-channel wireless device while performing basketball free throw and pass-catching tasks. The results of the repeated-measures analysis of variance on spectral power density indicated higher cortical activation in the elite group across all frequency bands (delta, theta, alpha, beta, and gamma). Furthermore, the cortical activation in the delta, theta, and alpha bands was higher in the free-throw task than in the pass-catching task. In most of the frontal sites, several central sites and one site in the parietal region had more cortical activity in the free-throw task than pass-catching in the alpha and theta bands. Findings show that elite subjects performed these tasks with optimized neural resource allocation, reduced cognitive load, and more automatically. Furthermore, enhanced cortical activity within frontal regions during free throw execution compared to pass-catching, coupled with the absence of significant parietal activity differences between these tasks, suggests functional interdependence between these neural processing systems specifically within the context of motor performance.

## Introduction

Selecting the most relevant stimuli in the physical world for processing, and filtering less relevant information allow us to quickly respond to critical changes in the environment and achieve more efficient behavioral goals [1]. In this field, theoretical approaches have different views on the importance and role of visual information in the production and control of movements [2]. Based on the ecological approach, perception is a bottom-up process. According to psychologist James J. Gibson, there is enough information in the environment in such a way that we can understand the world directly and with no need for interpretation [3–5]. On the contrary, according to cognitive approaches, perception is a top-down process. According to psychologist Richard Gregory, previous experience and knowledge about a stimulus help us infer [6,7].

Functional imaging, neurophysiological, and neuropsychological studies have provided evidence that frontoparietal networks greatly contribute to top-down and bottom-up sensory factors [8–13]. Furthermore, it has been reported that the bottom-up processing system is concentrated in the parietal cortex [12–14], and the top-down processing system is concentrated in the frontal cortex [11,12,15]. However, the occipital cortex has also been reported in attention studies [16].

The existence of electric currents in the brain was discovered by Richard Caton in 1875 [17]. About 54 years later, Hans Berger detected electrocortical activity on the surface of the human scalp [18,19]. Electroencephalography (EEG) has undergone enormous advances over the years [20]. This tool directly measures neural activity in real-time by evaluating the brain-behavior relationship. Despite the low spatial resolution regarding the origin of neural activity, it has a very high temporal resolution [21]. This tool facilitates a deeper understanding of the neural mechanisms underlying cognitive, sensory, and motor processes [21,22], especially for understanding top-down and bottom-up visual processing, by examining cortical activity patterns (e.g., [12,23–28]). Furthermore, it has been reported that expert executors use more appropriate cortical processing to perform a certain task than less-skilled executers, leading to better quality and more compatible motor output [29]. Therefore, it appears that evaluating the physiological mechanisms underlying the implementation of sports behavior is an important research topic in sports sciences [30,31]. Each sport has unique attentional characteristics, and to date, attentional control has been studied in three main categories of tasks: Targeting, interceptive timing, and tactical tasks [32,33]. During bottom-up processing, the athlete processes an immediate salient stimulus (e.g., an interceptive timing task), while in top-down processing, the athlete is guided by higher-level strategies that are developed over years of training and competition (e.g., a targeting task; [34]).

Numerous studies have evaluated neuroscience to interpret athletes’ performance in recent years [35]. These studies cover elite athletes with long-term sports experience and also compare them to groups with fewer skills, beginners, or non-athlete [31,36–43]. In the study of targeting and interceptive timing tasks (with less research literature), the neural activity in the seconds leading to the skill execution (especially 3–4 consecutive seconds) is examined during the task preparation and execution [23,31,42–48].

Studies examining changes in spectral power, particularly in the alpha band, during targeting tasks have revealed several key characteristics in elite athletes. A general increase in alpha band power is observed compared to beginner or non-athlete groups, suggesting a reduction in overall cerebral cortex activity and the inhibition of task-irrelevant information; this finding has been widely documented [37,40,43,49–51]. Furthermore, hemispheric asymmetry favoring the left hemisphere over the right is evident across the theta, alpha, and beta frequency bands in elite athletes, a phenomenon less pronounced or absent in novices [37,43,50]. Finally, spectral power changes are shown to reflect the specific characteristics of the task itself [37,43,48]. For instance, during a golf putt, a decrease in alpha power in the second before the execution has been interpreted as a shift in the athlete’s mental state from relaxation to focus [48]. Conversely, in a basketball free throw, an increase in alpha power within the same timeframe suggests a shift from focus to a relaxed mental state [43]. These differences demonstrate that, while both activities fall under the targeting tasks category, they engage distinct cognitive processes [43]. Overall, the results obtained in comparisons of different tasks such as golf [52], karate [49], shooting [36,37,41], or basketball [43] have been contradictory and require further research.

Moreover, only a few studies have evaluated changes in cortical activity during the preparation and execution of interceptive timing tasks [44–46,53,54]. In this regard, an increase in theta power is seen after falling the ball compared to before falling [44], an increase in the theta band in the left hemisphere [46] and an increase in the beta band in the right hemisphere [45]. Additionally, decreases in low-alpha (8–10 Hz) and beta frequency bands have been reported during catching [55]. According to these reports, it is assumed that if skilled execution in a specific domain is associated with distinct EEG profiles, this can help us understand cortical processes underlying peak performance [56].

Despite the sports professionals’ demands to understand the brain behavior links and effective factors in sports performance, sports neuroscience is still in its early stages [57]. According to research literature, bottom-up and top-down processing are two different ways to understand stimuli [32,33]. Unfortunately, most studies have investigated targeting tasks (top-down processing) but have given little consideration to processing models. Other dimensions, such as bottom-up processing in interceptive timing tasks, and a comparison of these two tasks and their processing paths, have been understudied. On the other hand, non-sports literature has reported that top-down influences are primarily mediated by low-frequency oscillations, particularly alpha and beta [24]. These studies generally implicate the theta band in both processing models and the gamma band in bottom-up processing [24,27,28,58,59]. However, very few studies have examined motor tasks from a processing systems perspective and reported that theta, especially midfrontal theta, is associated with top-down control functions [23,25,26]. Additionally, the differences in activation levels and cortical areas of elite people from other groups (beginners or non-athletes) double the need to investigate this issue. Even though visual spatial attention has been investigated by various technologies and methods for years, its neural mechanisms have not been well understood [60]. Due to the lack of standard methods used in sports and EEG and the lack of consensus on the physiological importance of various frequencies recorded for exercise [20], studies have had a low effect on our understanding of the neural basis of high-level performance [61]. Most EEG studies in sports sciences have largely focused on alpha power but neglected other spectral powers in this field. These studies have agreed on high alpha in optimal performance. However, a constant increase in alpha has not been associated with improved performance in the sports literature [38,48]. Furthermore, studies have measured alpha power in a few numbers of recording sites, and the fact that changes in specific frequency bands do not occur separately has been neglected [21]. A few studies have been conducted using EEG during actual sports activity. If we measure human cognition in laboratories, the results improve our knowledge for those certain conditions, but it cannot be necessarily generalized to real-world environments [62] and has no great effect on experts at least partially due to the lack of ecological validity and task representativeness [21]. Moreover, the use of spatial filtering techniques such as independent component analysis (ICA) and the combination of this algorithm with other algorithms such as artifact subspace reconstruction (ASR) can be applied to EEG data, leading to a more appropriate performance to limit motion artifacts [63]. The present sought to investigate the effect of targeting (basketball free throw) and interceptive timing (pass catching) tasks on the brain waves (delta, theta, alpha, beta, and gamma bands) of elite and educated basketball players in the seconds before and during the tasks, using new mobile techniques that have more ecological validity and provide the opportunity to evaluate the interactions between cognition and sports behavior in real-world environments.

## Experimental procedures

### Sample

Twenty participants voluntarily enrolled in this study. The sample size was determined a priori using G*Power software, with each group requiring ten participants (effect size: 0.7, alpha level: 0.05, power: 0.95). Ten participants were elite basketball players who met all inclusion criteria: (1) eligibility for national and premier league teams; (2) a minimum of five years of professional training; and (3) an average of more than three two-hour training sessions per week over the past two years. The remaining ten participants comprised the educated group. Participants in the educated group had at least two years of collegiate basketball experience and reported an average of 1.5 hours of weekly practice. All participants had normal vision [64] and reported no history of neurological or psychiatric disorders. To control for physiological variables, all participants were instructed to obtain eight hours of sleep the night before the experiment and to avoid caffeine, nicotine, and all medications. Subjects were unambiguously right-handed according to the Edinburgh Handedness Inventory [65]. Participants were fully informed verbally and in writing about the nature and demands of the study. They completed a health history questionnaire and were informed that they could withdraw from the study at any time, even after giving their written consent. The protocol of this study was approved by ethics committee of Shahid Beheshti University of Faculty of Sports and Health Sciences and is in conformity with the declaration of Helsinki (approved number IR.SBU.REC.1400.167). The date range for participant recruitment was from September 26, 2021 to November 24, 2021.

### Equipment

EEG signal acquisition was performed using the g.Nautilus mobile wireless EEG system (g.tec, [66]). This CE-certified device allows for 32-channelEEG recordings with 24-bit resolution and a 500 Hz sampling rate. The device records EEG data in real-time and transmits it via Bluetooth from a module placed at the back of the head to a Bluetooth-enabled module connected to a laptop via USB. Thirty-two EEG sites were monitored, corresponding to the following locations in the international 10–20 system: FP1, FP2, AF3, AF4, F3, Fz, F4, F7, F8, FC1, FC2, FC5, FC6, C3, Cz, C4, T7, T8, CP1, CP2, CP5, CP6, P3, Pz, P4, P7, P8, PO3, PO4, Oz, PO7, and PO8. AFz served as the ground electrode, and A2 (right earlobe) served as the reference. EEG data analysis was performed using MATLAB (R2022b) and the EEGLAB toolbox (v2021.0). Raw EEG data were imported into EEGLAB using the method described on the EEGLAB website (https://eeglab.org/). The data were received in MATLAB Simulink through a block provided by g.tec and saved in MATLAB (.mat) format, forming a crucial step in the data processing pipeline. At the beginning and end of each trial, a mouse trigger was added within the MATLAB environment; this trigger was simultaneously recorded with the EEG signals. EEG data were filtered using a 1651-order Finite Impulse Response (FIR) bandpass filter (pop_eegfiltnew in EEGLAB) with a passband of 1–40 Hz (-6 dB cutoff). Independent component analysis (ICA) was performed, with artifact identification guided by the ADJUST MATLAB plugin and supplemented by visual inspection of the time and frequency domains, scalp topographies, and raster plots. Subsequently, artifact subspace reconstruction (ASR) was applied to further suppress movement artifacts. The combination of ICA and ASR has been shown to be more effective in mitigating movement-related artifacts than ICA alone [63]. Power spectral density (PSD) analysis was performed using Welch’s method. Research suggests that top-down effects are primarily mediated by low-frequency oscillations, particularly alpha and beta [24]. These studies often implicate the theta band in both processing models, while the gamma band is involved in bottom-up processing [24,27,28,58,59]. The delta band has also been implicated in interference control, which inhibits sensory urges that interfere with task performance [67]. Therefore, the present study investigated the behavior of these bands and calculated the absolute power in different frequency bands, including delta (1–4 Hz), theta (4–8 Hz), alpha (8–12 Hz), beta (13–30 Hz), and gamma (30 Hz and above).

### Procedure

The session started with a 5-minute warm-up period (including dribbling, receiving passes, and basketball free throws). After the cap was secured on the participant’s head, conductive electrode gel was injected into the electrodes with disposable syringes and needles. Before recording the EEG signal, the impedance levels of the electrodes were adjusted to below 5 kΩ, and the quality of the signals was checked. Using a standard adult men’s basketball (size 7 with a circumference of 29.5 inches), the EEG data of the subjects was recorded during the basketball free throw and pass-catching for 5 trials. Participants were instructed to prepare, take their time, and perform when ready. They were encouraged to do their best. Following five free throws and a 1-minute rest period to mitigate fatigue and maintain concentration, the participants performed five pass-catching trials. Participants were not allowed to dribble or touch the floor between free throw and pass catching tasks. The basketball hoop was 10 feet (305 cm) above the ground. Basketball free throw test: The subject stood behind the throw line at a distance of 15.09 feet)406 cm) from the basket and performed the throwing task after receiving an audio stimulus. Pass-catching test: With the start of the audio stimulus, the training assistant threw the ball from a distance of 5 meters at the height of the subject’s chest and the player caught the ball. Simultaneously with the audio stimulus, a manual mouse trigger was pressed on the computer by the researcher. At the same time, the video of the task performances was recorded. The video and audio stimulus were matched with the trigger and recorded EEG data. For the execution of each trial, 4 seconds were recorded from the moment of the trigger signal to the complete execution of the task. After removing the first second (the possible distractive effect of the sound signals on the subjects), a total of 3 seconds were used for analysis (S1 Dataset). The data from the start of the visible movement until the execution of the tasks (free throw/ pass-catching) for 1 second, is considered as the execution of the task (time 3). Also, the data of 2 seconds before the start of execution were considered as 1 second before execution and 2 seconds before execution (time 1 and time 2).

### Statistical analysis

All statistical analyses were performed using the SPSS version 27.0 software package. The Shapiro-Wilk normality test was used to evaluate whether continuous variables exhibited a normal distribution. All analyzes were based on logarithmically transformed absolute spectral power values. The power values of each frequency band were analyzed in a repeated measure ANOVA 2 (group: Elite/Educated) × 3 (time: 1,2,3) × 2 (task: basketball free throw/pass-catching). When the rule of sphericity was violated, the Greenhouse-Geisser correction was conducted. T-test and Tukey’s post hoc test was also employed to compare the levels with a significant difference (*p *≥ 0.05).

## Results

The results are presented below separately for 5 frequency bands (alpha, theta, beta, gamma, and delta).

### Delta band

**Fig 1:**
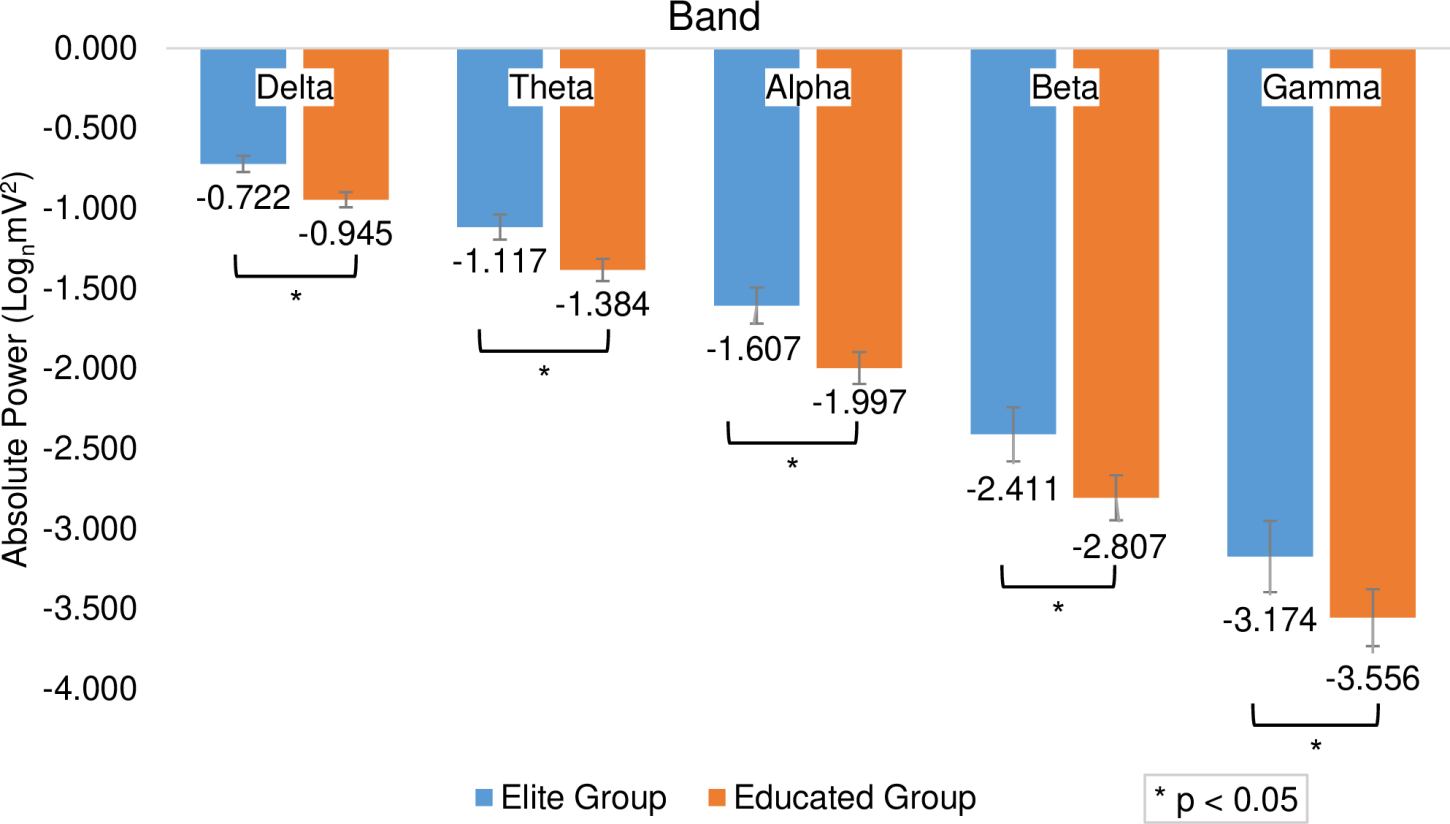
The average scores of subjects’ cortical activity in different band based on elite and educated groups. The asterisk indicates a significant difference between the two groups.

The repeated measures ANOVA test results indicated a significant main effect of the group variable (F_(1,196)_ = 6.762, *p*_* *_= 0.010, η^2^ = 0.033), showing that the elite group (M = -0.722, SD = 0.741) had higher scores than the educated group (M = -0.945, SD = 0.686, d = 0.313; Fig 1). These results also indicated a significant main effect of the task variable (F_(1,196)_ = 11.237, *p*_* *_< 0.001, η^2^ = 0.054), showing that free-throw task (M = -0.689, SD = 0.667) had significantly higher scores than the pass-catching task (M = -0.978, SD = 0.747, d = 0.708). Furthermore, the interaction between the task and the group was not significant (F_(1,196)_ = 1.218, *p *= 0.271, η^2^ = 0.006), indicating no difference between the two tasks separately in groups.

These results indicated there was no difference between different times (F_(2, 392)_ = 0.819, *p *= 0.442, η^2^ = 0.004). The main effect of the site (F_(31, 6076)_ = 126.122, *p *< 0.001, η^2^ = 0.392) and the interaction of site and group were significant (F_(31, 6076)_ = 3.775, *p *< 0.001, η^2^ = 0.019), indicating that the elite group had higher scores than the educated group in Fp2, F4, F7, Fz, F8, Fc5, Fc1, C3, Cz, Cp5, Cp1, Cp6, P7, P3, P4, Po4, Po3, and Oz (*p *< 0.05, Cohen’s d ranged from 0.217 to 0.442) but there was no difference between in other sites (*p *> 0.05). Furthermore, two tasks had significant difference in sites (F_(31, 6076)_ = 2.097, *p *= 0.005, η^2^ = 0.011). According to further study, the free-throw task had a significant priority over the pass-catching task in most areas (*p *< 0.05, Cohen’s d ranged from 0.207 to 0.488) except for F3, Fp2, T7, Po3, and Po8 in which there was no difference between the two tasks (*p *> 0.05).

### Theta band

**Fig 2:**
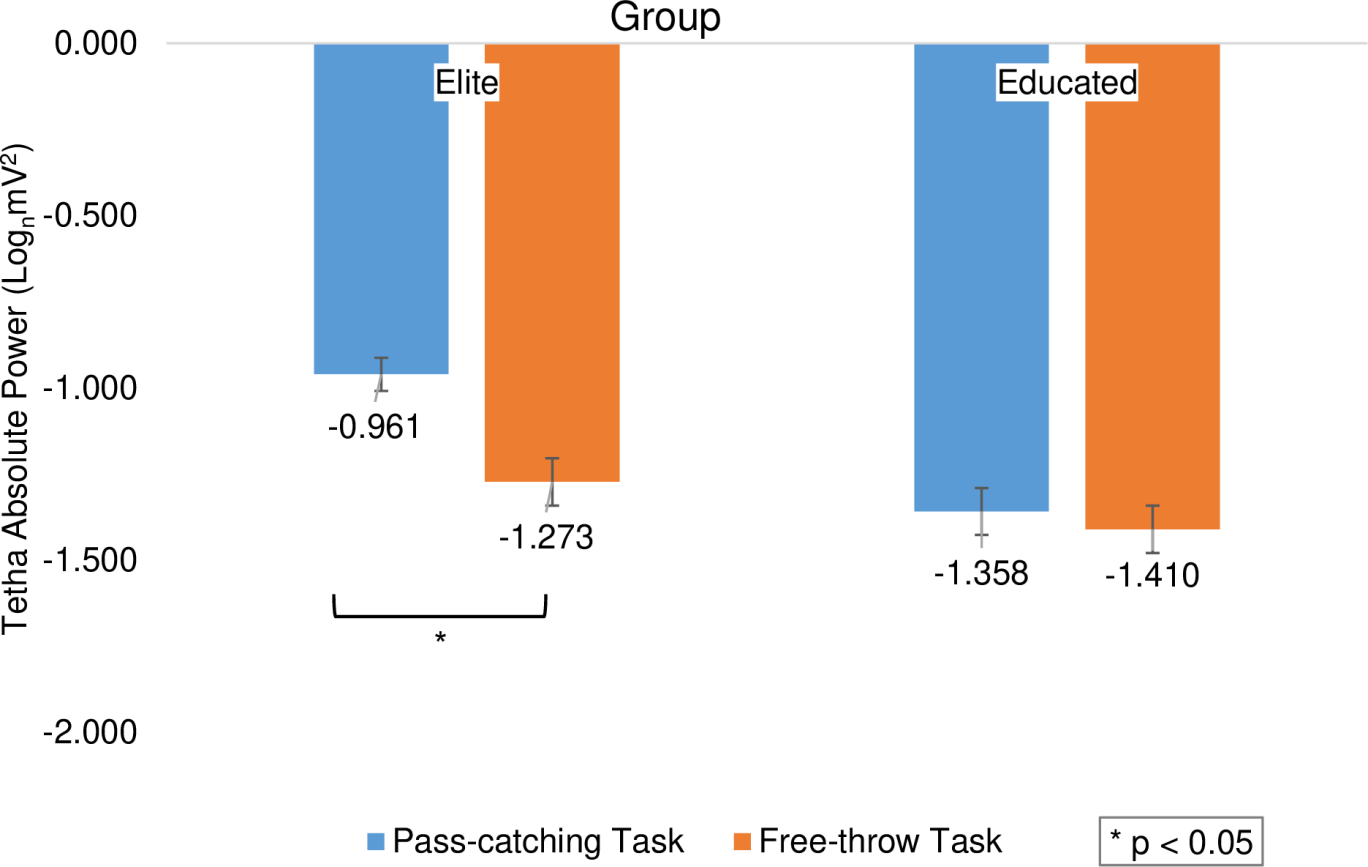
The average scores of subjects’ theta band activity based on elite and educated groups in free-throw and pass-catching tasks. The asterisk indicates a significant difference between the two tasks.

The repeated measures ANOVA test results indicated a significant main effect of the group variable (F_(1,196)_ = 18.468, *p *< 0.001, η^2^ = 0.086), showing that the elite group (M = -1.117, SD = 0.538) had significantly higher scores than the educated group (M = -1.384, SD = 0.489, d = 0.520). There was significant main effect in task (F_(1,196)_ = 8.518, *p *= 0.004, η^2^ = 0.042) and the free-throw task (M = -1.159, SD = 0.494) had higher scores than the pass-catching task (M = -1.341, SD = 0.551, d = 0.347). Furthermore, two tasks had significantly different scores in the groups (F_(1,196)_ = 4.362, *p *= 0.038, η^2^ = 0.022). Based on the follow-up studies in the elite group, the free-throw task (M = -0.961, SD = 0.444) had higher scores than the pass-catching task (M = -1.273, SD = 0.580, *p *< 0.05, d = 0.604) but this difference was not observed in the educated group (*p *> 0.05; Fig 2).

**Fig 3:**
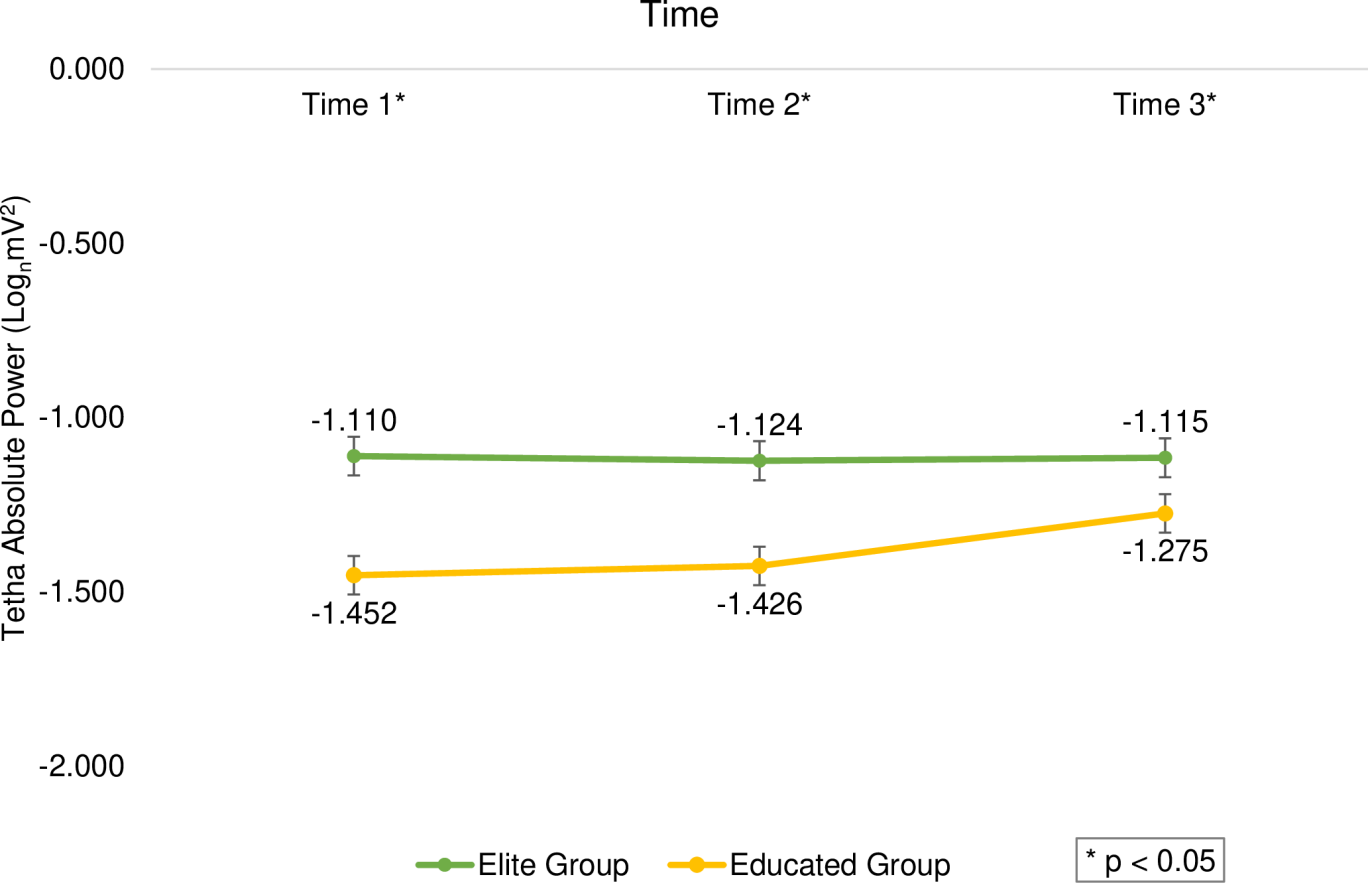
The average scores of subjects’ theta band activity based on elite and educated groups in 3-time windows. The asterisk indicates a significant difference between the two groups.

There was a significant main effect between times (F_(2, 392)_ = 5.249, *p *= 0.006, η^2^ = 0.026), and follow-up analyses indicated that time 3 (M = -1.195, SD = 0.572) received higher scores than the time 1 (M = -1.281, SD = 0.521, *t*_(199)_ = -2.569, *p *= 0.011, d = 0.182) and time 2 (M = -1.275, SD = 0.496, *t*_(199)_ = -2.513, *p *= 0.013, d = 0.178). The results also indicated a significant difference between the two groups at different times (F_(2, 392)_ = 5.234, *p *= 0.006, η^2^ = 0.026), and the elite group had a significant priority over the educated group in all three times (*p *< 0.05, Cohen’s d ranged from 0.281 to 0.693; Fig 3). Furthermore, there was no significant difference between the two tasks at different times (F_(2, 392)_ = 2.467, *p *= 0.086, η^2^ = 0.012).

These results indicted a significant main effect in sites (F_(31, 6076)_ = 146.486, *p *< 0.001, η^2^ = 0.428) also, there was a significant difference between the two groups in different sites (F_(31, 6076)_ = 4.872, *p *< 0.001, η^2^ = 0.024), and the elite group had higher scores than the educated group in most areas (*p *< 0.05, Cohen’s d ranged from 0.224 to 0.708) except for Af3, Cp2, Pz, and Po7, in which there was no difference between the groups (*p *> 0.05). Furthermore, there was a significant difference between the two tasks in different sites (F_(31, 6076)_ = 2.648, *p *< 0.001, η^2^ = 0.013). According to further study, the free-throw task had a significant priority over the pass-catching task (*p *< 0.05, Cohen’s d ranged from 0.244 to 0.529) in most areas except for F3, Fc5, T8, Cp1, Cp6, P7, P3, Pz, Po4, Fp2, T7, Po3, Oz, and Po8 areas in which there was no difference between the two tasks (*p *> 0.05). Finally, there were significant differences in the interaction between the task, group and the site (F_(31, 6076)_ = 4.212, *p *< 0.001, η^2^ = 0.021). According to further tests, the free-throw task had higher scores in the elite group in most areas (*p *< 0.05, Cohen’s d ranged from 0.348 to 0.840) except for in Cz, T8, Cp1, Cp6, P7, P3, Pz, T7, and Po3 areas where there was no difference between the two tasks (*p *> 0.05). In the educated group, the free-throw task in the C4, fz, f4, fc1, c3 and cz area (*p *< 0.05, Cohen’s d ranged from 0.302–0.347), and the pass-catching task in the F3 area (*p *< 0.05, d = 0.321) had higher scores, but there was no difference between the two tasks in other areas (*p *> 0.05).

### Alpha band

**Fig 4:**
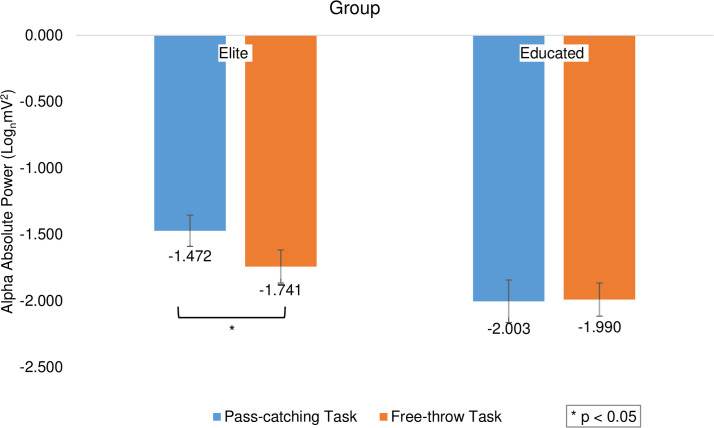
The average scores of subjects’ alpha band activity based on elite and educated groups in free-throw and pass-catching tasks. The asterisk indicates a significant difference between the two tasks.

The repeated measures ANOVA results indicated a significant main effect of the group variable (F_(1,196)_ = 46.464, *p *< 0.001, η^2^ = 0.192), showing that the elite group (M = -1.607, SD = 0.496) had higher scores than the educated group (M = -1.997, SD = 0.503, d = 0.780). There was a significant main effect in the task variable (F_(1,196)_ = 5.012, *p *= 0.026, η^2^ = 0.025) and the free-throw task (M = -1.738, SD = 0.536) had higher scores than the pass-catching task (M = -1.866, SD = 0.529, d = 0.240). Furthermore, the interaction between the task and the group was significant (F_(1,196)_ = 6.069, *p *= 0.015, η^2^ = 0.030). Based on the follow-up studies in the elite group, the free-throw task (M = -1.472, SD = 0.475) had higher scores than the pass-catching task (M = -1.741, SD = 0.482, *p *< 0.001, d = 0.562) but this difference was not observed in the educated group (*p *> 0.05; Fig 4).

**Fig 5:**
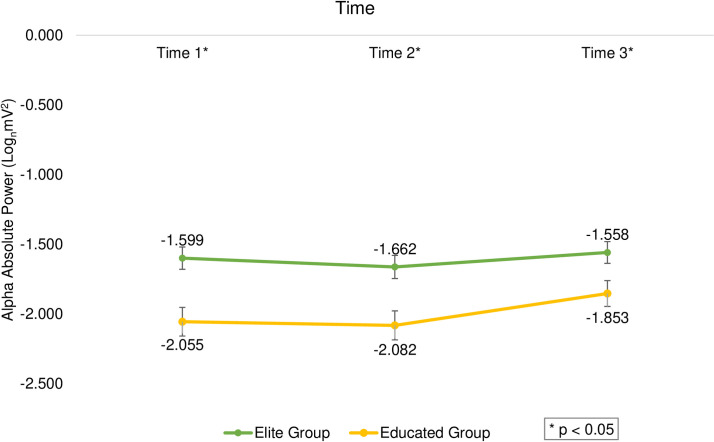
The average scores of subjects’ alpha band activity based on elite and educated groups in 3-time windows. The asterisk indicates a significant difference between the two groups.

**Fig 6:**
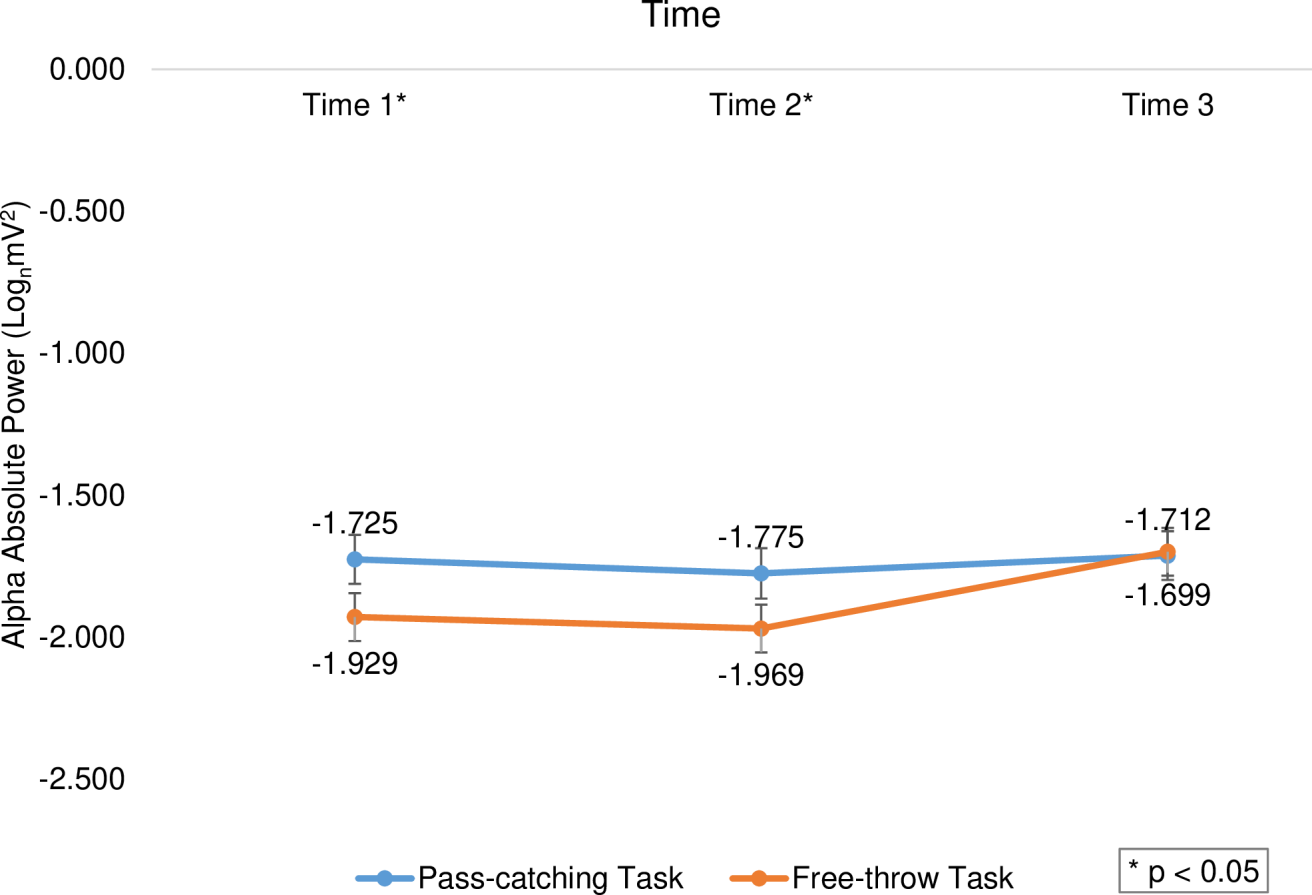
The average scores of subjects’ alpha band activity in free-throw and pass-catching tasks in 3-time windows. The asterisk indicates a significant difference between the two tasks.

The results indicated a significant main effect of time (F_(2,391)_ = 13.696, *p *< 0.001, η^2^ = 0.065), and follow-up analyses indicated that time 3 (M = -1.706, SD = 0.576) received higher scores than the time 1 (M = -1.827, SD = 0.521, *t*_(199)_ = -3.182, *p *= 0.002, d = 0.225) and time 2 (M = -1.872, SD = 0.497, *t*_(199)_ = -4.585, *p *< 0.001, d = 0.324). There was a significant difference between the two groups at different times (F_(2,391)_ = 3.316, *p *= 0.037, η^2^ = 0.017; Fig 5) and the elite group had a significant priority over the educated group in all three times (*p *< 0.001, Cohen’s d ranged from 0.527 to 0.973). Furthermore, there was a significant difference between the two tasks at different times (F_(2,391)_ = 6.927, *p *= 0.001, η^2^ = 0.034), and the free-throw task had higher scores in times 1 and 2 (*p *< 0.05, Cohen’s d ranged from 0.396 to 0.397), but there was no difference between the two tasks in time 3 (*p *> 0.05; Fig 6).

These results indicted a significant main effect in site (F_(31,6076)_ = 127.976, *p *< 0.001, η^2^ = 0.325), also the interaction of site and group (F_(31,6076)_ = 5.560, *p *< 0.001, η^2^ = 0.028), showing that the elite group had higher scores than the educated group in most areas (*p *< 0.001, Cohen’s d ranged from 0.356 to 0.907) except for Af3 and Pz sites in which there was no difference between the groups (*p *> 0.05). Furthermore, there was a significant difference between the two tasks in different sites (F_(31,6076)_ = 3.915, *p *< 0.001, η^2^ = 0.020). According to further study, the free-throw task had a significant priority over the pass-catching task (*p *< 0.05, Cohen’s d ranged from 0.232 to 0.513) in most frontal areas, several central sites, and an electrode from the right parietal (Af3, Af4, F7, Fz, F4, F8, Fc1, Fc2, Fc6, C3, C4, Cp5, Cp2, and P4), but there was no difference between the two tasks in other areas (*p *> 0.05). Finally, there were significant differences between group and tasks in several sites (F_(31,6076)_ = 4.660, *p *< 0.001, η^2^ = 0.023), according to further study, the free-throw task had higher scores (*p *< 0.05, Cohen’s d ranged from 0.288 to 0.801) in the elite group in most areas except for in Cz, T8, Cp1, Cp6, Pz, Po4, Fp2, T7, Po3, Oz, Po7, and Po8 areas where there was no difference between the two tasks (*p *> 0.05). In the educated group, the free-throw task in the C4 and F4 area (*p *< 0.05, Cohen’s d ranged from 0.273 to 0.338), and the pass-catching task in the Fp1, Af3, F3, Fc5, and T7 areas (*p *< 0.05, Cohen’s d ranged from 0.303 to 0.456) had higher scores but there was no difference between the two tasks in other areas (*p *> 0.05).

### Beta band

The repeated measures ANOVA results revealed a main effect of group (F_(1,196)_ = 54.358, *p *< 0.001, η^2^ = 0.217), showing that the elite group (M = -2.411, SD = 0.405) had significantly higher scores than the educated group (M = -2.807, SD = 0.479, d = 0.891; Fig 1). There was no significant main effect in task variable (F_(1,196)_ = 0.162, *p *= 0.687, η^2^ = 0.001).

These results indicated significant main effect in time variable (F_(2, 392)_ = 7.340, *p *< 0.001, η^2^ = 0.036), also time 3 (M = -2.553, SD = 0.543) showed higher scores than time 2 (M = -2.651, SD = 0.438, *t*_(199)_ = -3.332, *p *= 0.001, d = 0.236); but there was no significant interaction between groups and times (F_(2, 392)_ = 1.243, *p *= 0.290, η^2^ = 0.006).

These results also indicated a main effect between sites (F_(31, 6076)_ = 175.588, *p *< 0.001, η^2^ = 0.473) and the interaction of group and sites (F_(31, 6076)_ = 5.157, *p *< 0.001, η^2^ = 0.026), analyses show that elite group had higher scores than the educated group in most sites (*p *< 0.05, Cohen’s d ranged from 0.290 to 1.059) except for Af3 and Pz sites in which there was no difference between the groups (*p *> 0.05).

### Gamma band

The repeated measures ANOVA results revealed a main effect in group variable (F_(1,196)_ = 40.739, _*p *_< 0.001, η^2^ = 0.172), showing that the elite group (M = -3.174, SD = 0.439) had higher scores than the educated group (M = -3.556, SD = 0.530, d = 0.784; Fig 1). There was no significant main effect in task variable (F_(1,196)_ = 1.703, *p *= 0.193, η^2^ = 0.009).

These results indicated significant main effect in time variable (F_(2, 392)_ = 9.895, *p *< 0.001, η^2^ = 0.048), and analyses indicated that time 3 (M = -3.294, SD = 0.567) showed higher scores than time 1 (M = -3.406, SD = 0.497, *t*_(199)_ = -3.360, *p *< 0.001, d = 0.238) and time 2 (M = -3.395, SD = 0.494, *t*_(199)_ = -3.356, *p *< 0.001, d = 0.237), but there was no difference between time 1 and time 2 (*p *> 0.05). The results indicating no significant interaction between groups and time (F_(2, 392)_ = 1.794, *p *= 0.176, η^2^ = 0.009).

These results also indicated there were significant main effect between sites (F_(31, 6076)_ = 218.195, *p *< 0.001, η^2^ = 0.527) and the interaction of site and group were significant (F_(31, 6076)_ = 5.184, *p *< 0.001, η^2^ = 0.026), indicating that the elite group had higher scores than the educated group in most areas (*p *< 0.05, Cohen’s d ranged from 0.341 to 1.024) except for Fp1, Af3, T8, Pz, and Po7 sites in which there was no difference between the groups (*p *> 0.05).

## Discussion

The present study investigated the effect of targeting and interceptive timing tasks on the brain waves of 20 elite and educated athletes in the seconds before and during task execution. The results indicated higher cortical activation levels in the elite group than in the educated group across all spectral powers, confirming the findings of other studies [36,37,43,49–51,68,69]. The increased cortical activation observed in elite subjects demonstrates enhanced focus, optimized neural resource allocation, and an improved ability to ignore distractions [23,41,43]. This result is consistent with the Fitts and Posner model of skill acquisition, which posits that enhanced focus, optimized neural resource allocation, and reduced cognitive load are hallmarks of automaticity [70]. According to Ericsson [71], experts activate only the essential neural networks through practice. This finding aligns with the neural efficiency hypothesis, which suggests that experts optimize performance by suppressing task-irrelevant processes and enhancing task-relevant ones [29,72,73].

However, lower activity in the alpha band of elite groups is shown in many cognitive and motor tasks [74,75]. This obvious difference in alpha responses among different tasks may be related to the complexity of a real task versus a controlled laboratory task [76]. In a study by Cook et al. [48], skilled golfers had much lower theta, alpha, and beta powers than beginners. Since changes in EEG show the task specificity [41,43], it can be argued that the cortical characteristics of the free throw and pass-catching tasks are different from tasks such as golf putting in this study.

In general, cortical activation in the delta, theta, and alpha bands was higher in the free-throw task than in the pass-catching task, but there was no difference between the cortical activity of the two tasks in the beta and gamma bands. The high increase in theta band activity during the free-throw task is indicative of attentional allocation [23,26]. Even though the theta of the free-throw task was higher than the theta of a pass-catching task in the present study, non-sports studies considered the theta band involvement in both processing models [24,27,28,58,59,77]). Furthermore, it was hypothesized that higher alpha activity may reflect active top-down inhibition in task-irrelevant brain regions [78] and the suppression of visual area involvement to filter out irrelevant visual information [79]. According to these reports, it appears that the free-throw task, in the present study, had less involvement in the task-related processes and less interference in unnecessary areas of the cerebral cortex compared to the pass-catching task. Few studies on interceptive timing tasks also indicated lower alpha band activity in the pass-catching phase [55]. Accordingly, alpha band activity demonstrates distinct functional roles during free-throw and pass-catching tasks. Specifically, free throws are associated with increased alpha activity (indicating the use of fewer neural resources, lower cognitive load, and greater relaxation) and the suppression of task-irrelevant information. These findings align with previous research on the role of alpha activity in motor performance and cognitive load [41,43]. Conversely, pass-catching elicits decreased alpha activity (indicating a higher cognitive load and a state of heightened focus), suggesting that more neural resources are recruited for skillful execution. This modulation of alpha power is consistent with the interpretation that lower alpha power signifies enhanced focus, greater cortical engagement, and resource mobilization for task planning [48]. In contrast, non-exercise related studies employing short-term memory tasks have shown that alpha activity increases with increasing memory load [78,79]. This discrepancy with the present study may stem from the different nature and foundation of these two categories of research. Exercise tasks, unlike purely cognitive tasks, have a motor nature and require complex interaction between the motor and cognitive areas of the brain. Furthermore, considerable evidence exists in the sports literature showing that patterns of electrical brain activity during different tasks are task-specific [38,41,43,48]. Therefore, not only is there a difference between exercise and non-exercise studies, but also within exercise studies themselves, different tasks have shown different cortical activation and interpretations. These findings highlight the importance of considering the type and nature of the task when interpreting patterns of brain activity.

Non-sports studies indicate that the beta band is involved in top-down effects [24], and the gamma band is involved in bottom-up effects [24,28]. However, such an effect was not observed between targeting (top-down processing) and interceptive timing (bottom-up processing) tasks in the present study. A key weakness in sports science research is its over-reliance on alpha band EEG dynamics, neglecting other frequency bands. This may be because sports and motor tasks are often designed with moderate difficulty, allowing participants to perform them in a typical mental state without requiring intense mental effort. Consequently, researchers suggest that frequency bands like gamma and delta may not fully reflect cognitive processes during these tasks. Despite the limited attention given to delta band activity in sports research, a few non-sports studies suggest that delta band activity plays a role in interference control, suppressing sensory input that disrupts task performance [67]. In the present study, the free throw task elicited greater delta band cortical activation than the pass-catching task. This finding aligns with Harmony’s [67] report, suggesting that increased delta activity during basketball free throws may reflect greater suppression of distracting sensory input to minimize interference with the task.

The overall results indicated that there was no significant difference in theta, alpha, and gamma frequency band activity between the first and 2^nd^ second before task execution. However, the third second of task execution exhibited significantly higher cortical activity compared to both the first and second seconds. Additionally, the second of executing tasks had higher scores in the beta band than in the 2^nd^ second. However, there was no difference between the seconds in the delta band before and during the tasks. The studies on optimal timing for analysis during execution indicated that EEG profiles became more prominent as approached the execution time [23,31,37,43]. The present study confirmed these reports because the cortical activity increased in most frequency bands in both free throw and pass-catching tasks as the task execution phase approached. However, the lack of difference in periods in the delta band in the present study also confirmed the reason for the scarcity of studies that have used this frequency band as a measure of cortical activity [42]. Few studies have evaluated changes in cortical activity during the seconds up to the execution of interceptive timing tasks [44–46,53,54]. Meanwhile, there was an increase in theta band after falling the ball compared to before falling [44,53] and an increase in theta power in the catching phase [55]. Studies also reported lower alpha and beta power during the catching phase [55]. The results of these few reports were to some extent contradictory with the present study because, in the present study, the researchers found that alpha and beta frequency bands increased along with the theta band in the pass-catching task as the execution phase approached. As it was found that EEG profiles were task-specific regarding the targeting tasks [38,41,43,48], it seems that the contradiction in interceptive timing tasks also indicated different characteristics.

The results in the theta and alpha bands indicated higher cortical activation in the elite group compared to the educated group at all three times. Furthermore, no differences were observed between the groups in the beta, gamma, and delta bands at all three times. The increased alpha band activity in the elite group compared to the educated group (at different times) is consistent with the concept of lower cerebral cortex activation (higher alpha power). This finding suggests an automatic stage of motor skill learning, as described by Fitts and Posner [70]. Conversely, the increased cortical activity (lower alpha power) and greater use of neural resources in the educated group suggest that these subjects executed tasks with a broader focus, likely corresponding to the focus stage in the skill acquisition model [70]. These results are consistent with previous research showing that higher alpha band activity in the second before execution correlates with fewer errors [52]. Machado et al. [80] also reported an increase in alpha band power during the catching phase, which they interpreted as indicative of a reduced demand for motor planning and preparation.

It is reported that skilled golfers have less cortical activity than beginners 2–3 seconds before execution, but a significant decrease in alpha power is observed in the last second before execution. According to these findings, the athletes’ mental state changes from relaxation to focus before the execution [48]. In the present study, it appears that the elite athletes’ mental state changes from focus to relaxation. As mentioned in different sections of this manuscript, EEG changes indicate the task characteristics [41]; hence, these characteristics are different from the characteristics of the golf putting task in this research. Furthermore, the reduction of alpha power in the pass-capturing phase in a study by Tombini et al. [55] proves the discovery of task-specific EEG profiles in the present study because despite the findings of Tombini et al. [55], an increase in cortical activity in the alpha band is observed as the pass-catching phase approaches in the present study.

In the present study, the higher theta power of the elite group than in the educated group indicates that the elites performed the skill in the last second with more attentional focus and optimal timing [23,26]. According to Doppelmayr et al., [23] elites and non-elites probably utilize different strategies during the preparation period, and elites can focus their attention correctly on the moment of skill execution while people with lower skill levels (e.g., educated) maintain a constant level of attention to goals [23]. A meta-analysis by Fang et al. [42] indicates that athletes create sufficient abilities and efficient strategies to allocate more attention resources.

In the alpha and theta bands, the free-throw task had more cortical activity than the pass-catching task in most prefrontal and frontal cortical areas, several central areas, and the P4 electrode in the right parietal, but there was no difference between the two tasks in other areas. In the delta band, the free-throw task had more cortical activity than the pass-catching task in most areas. As mentioned earlier, higher alpha may reflect active top-down inhibition in task-irrelevant brain regions [78]., and non-involvement of visual regions to suppress the irrelevant visual information processing [79]. According to these reports, it appears that the free-throw task had less interference with the task-related process in the present study, indicating less interference from unnecessary areas of the cerebral cortex compared to the pass-catching task. Furthermore, studies indicate a close relationship between frontal theta and cognitive control [81,82]. When a task becomes challenging, much attention to it leads to stronger activity in the frontal region [42]; hence, it appears that the higher theta activity in the basketball free-throw task is the outcome of increasing the complexity of this task compared to the pass-catching task. In the present study, the cortical activity of the delta band was higher in the free-throw task than in the pass-catching task. According to Harmony’s report [67], the activity in this band probably led to greater inhibition of distracting factors for less interference with the free-throw task.

According to the researchers’ report, the top-down processing system of visual perception is concentrated in the frontal and prefrontal cortex [83,84] and is preferentially activated during tasks that require selecting a stimulus from distracting stimuli [85]. It is reported that the cerebral cortex uses the Parietal region in bottom-up processing because it has to make judgments based on the sensory qualities of the stimuli [86]. However, other researchers reported a significant increase in cortical activity in top-down processing in comparison to bottom-up processing in the frontal region, but there was no continuous increase in the parietal region [12]. In the present study, the alpha and theta bands’ activity during the execution of the basketball free-throw task (top-down processing) was higher than the pass-catching task (bottom-up processing), but this difference was only observed in the P4 electrode in the parietal region. This difference was in favor of top-down processing (i.e., basketball free-throw task).

Despite the consistency of many non-sports studies that the bottom-up processing was focused on the parietal region, the present study did not find any difference between basketball free-throw and pass-catching tasks. Therefore, it appears that both tasks used the neural resources of the parietal region to the same extent in the present study. The activity of the P4 electrode was an exception as it was higher in the free-throw task than in the pass-catching task. The activation of the P4 electrode in the parietal region might be for maintaining attention and achieving optimal performance in the free-throw task because the alpha of the P4 electrode was involved in attracting attention by inhibiting irrelevant information [87]. Therefore, the increase in parietal alpha power prevented the reorientation of attention to task-irrelevant stimuli during target-oriented behavior [88]. The results suggest that the relationship between these two processing systems is more nuanced than solely attributable to their structural differences. These systems demonstrate functional interdependence, particularly in the execution of motor tasks. While non-sport studies, grounded in the paradigm of frontal/prefrontal involvement in top-down processing and parietal involvement in bottom-up processing, have consistently supported this framework, the present study reveals a somewhat discrepant pattern. This discrepancy can be primarily attributed to two key factors: 1) the motoric nature of the tasks employed in this study, in contrast to the predominantly non-motor tasks and controlled conditions of prior research; and 2) the unique task-dependent pattern of frequency band activity observed in the present investigation. Prior research within the field of exercise neuroscience has consistently demonstrated a significant influence of task type on the activity patterns observed within these specific frequency bands.

Since sports neuroscience studies should stimulate real-world sports settings to bridge the gap between artificial stimuli and actual sports situations, the present study aimed to investigate the brain activity of basketball players during simulated tasks using a portable EEG device. Conducting this study in a world-class sports environment allowed us to gain a more accurate understanding of the interaction between cognitive, motor, and environmental factors during athletic performance. Additionally, by employing a combination of advanced data analysis methods such as ICA and ASR, we were able to reduce motion-related noise and extract brain activity signals with greater precision. This enables us to generalize our findings to real-world sports settings with more confidence. However, by integrating multi-modal data streams (e.g., electromyography, electroencephalography and eye tracker) and utilizing advanced signal processing techniques in future studies, we can overcome many limitations, including more accurate interpretation of results. This approach will be crucial when facing the challenge of separating cortical and subcortical contributions during dynamic task paradigms. Sports such as volleyball, handball, soccer, tennis, and basketball, among others, necessitate a combination of targeting and an interceptive timing task. Therefore, expanding research into visual processing models in these sports can offer novel insights into the intricate cognitive and neural underpinnings of elite athletic performance. We hope that the results of this research can be an effective step in advancing sports goals for athletes and coaches. The results of this research can also be used in neurofeedback protocols.

## Supporting information

S1 Dataset Data file used for statistical analysis, provided in Excel format. (XLSX)
